# Supplementary material for: Determinants of undernutrition and overnutrition among reproductive-age women in Bangladesh: Trend analysis using spatial modeling
Source: PLoS One. 2023 Jul 18;18(7):e0282998. doi: 10.1371/journal.pone.0282998 (PMC10353797; doi:10.1371/journal.pone.0282998)
Supplement: Attachment — Submitted filename: Comments.docx [file pone.0282998.s001.docx]

This is an important area of research in Bangladesh with good sample size to support the study findings. There are important concerns with that needs to be address, however, that require major revision.

Authors may consider following suggestions for revising the manuscript:

Comment: The missing of page number and line number becomes difficult for the reviewers to mention the place for making the comments and suggestion.

Comment: It can also be mentioned that why traditional linear regression models may fail to capture the spatial effects and why you choose Bayesian Geo-additive model. Very briefly, it can also be highlighted about the importance of accounting for spatial autocorrelation and how it can lead to bias in estimates.

Comment: It will be interesting if the authors perform the analysis by splitting the spatial effects into structured and unstructured spatial effects.

Comment: The authors must perform several model diagnostic tests to arrive at the best model, and incorporate the model selection parameters in the manuscript.

I would suggest reading the paper by Marbaniang et al., (2022) on model diagnostic and spatial effect.

Comment: It is advisable to use two more models here to understand the influence of spatially structure and spatially unstructured heterogeneity. In this way it can be known about the drop/increase in DIC

Comment: Instead of using wealth index as a linear effect. A continuous variable “wealth score” can be used to explore the non linear effect.

Comment: You need to mention how you are defining your neighbor here? Did you use adjacency metric or any other method to define neighborhood, then you need to mention about it and may write the equation/formula for the same
