# Supplementary material for: Determinants of undernutrition and overnutrition among reproductive-age women in Bangladesh: Trend analysis using spatial modeling
Source: PLoS One. 2023 Jul 18;18(7):e0282998. doi: 10.1371/journal.pone.0282998 (PMC10353797; doi:10.1371/journal.pone.0282998)
Supplement: Attachment — Submitted filename: Comments2.docx [file pone.0282998.s002.docx]

I am satisfied with the response of the authors as they have incorporated all the comments and suggestions. However, I would suggest the authors to cite the related paper by Marbaniang et al., (2022) as it is related to Bayesian Technique used in your paper.

I wish the authors all the best and I recommend the paper for publication.
